# HOPE and AMPK activation reduce reperfusion injury and metabolic dysfunction in primate steatotic liver grafts

**DOI:** 10.1038/s41598-025-96265-3

**Published:** 2025-04-06

**Authors:** Tao Li, Pengkang Chang, Yimeng Wang, Yihong Song, Pengxiang Qu, Bo Wang, Yi Lyu, Liangshuo Hu

**Affiliations:** 1 Department of Hepatobiliary Surgery, Institute of Advanced Surgical Technology and Engineering, The First Affiliated Hospital of Xi’an Jiaotong University, Xi’an, China; 2 Department of Urology, Urologic Surgery Center, Xinqiao Hospital, Third Military Medical University (Army Medical University), Chongqing, China; 3 The First Affiliated Hospital of Xi’an Jiaotong University, Xi’an, China; 4 Laboratory Animal Center, Xi’an Jiaotong University Health Science Center, 76 Yanta West Road, Xi’an, 710061 Shaanxi China

**Keywords:** Hypothermic oxygenated perfusion (HOPE), Steatotic liver grafts, Living liver transplantation, AMPK activator, Primate models, Liver diseases, Hepatology

## Abstract

Living liver transplantation has become a significant and evolving aspect of organ transplantation, with a notable proportion of cases involving pediatric patients. Metabolic-associated fatty liver disease (MAFLD) is the most prevalent chronic liver disease. The growing number of individuals with MAFLD has led to an annual increase in the proportion of non-alcoholic fatty liver donors for pediatric living liver transplantation. Hypothermic oxygenated perfusion (HOPE) has been demonstrated to improve graft quality through the implementation of a continuous mechanical perfusion cycle. However, there is currently a paucity of evidence regarding its ability to reduce steatosis and improve prognosis within a shorter time window of living-organ transplantation, especially in primate models. This study simulated steatotic liver grafts in living liver transplantation using the MAFLD model of the cynomolgus monkey and explored the effects of HOPE combined with the AMPK activator AICAR on the amelioration of the donor liver. The left outer lobe livers were statically cold preserved for two hours, subjected to HOPE for two hours, or treated with HOPE + AICAR (1 mmol/L) for two hours, respectively. Subsequently, a normothermic ex vivo reperfusion model (IRM) simulating post-transplant reperfusion was established using diluted autologous blood. Following simulated reperfusion in vitro, steatotic liver grafts in the static cold preservation group exhibited notable reperfusion injury. The degree of reperfusion injury induced by the remaining two groups was reduced, with the HOPE + AICAR group showing the most significant reduction (*P* < 0.05). The adenosine triphosphate (ATP) level of the hepatic tissues in the HOPE + AICAR group was observed to improve at two hours of reperfusion, exhibiting a significantly higher level than that in the cold-preserved group (*P* < 0.05). Furthermore, the HOPE + AICAR group exhibited a notable decline in MDA levels (*P* < 0.05), accompanied by a considerable reduction in 8-OHdG and lactate concentrations in both the liver tissue and perfusate. Additionally, there was a marked decrease in the release of TNF-α and IL-6 cytokines, along with a reduction in TLR-4 activation (*P* < 0.05). In comparison to the cold-preserved and HOPE groups, the HOPE + AICAR group demonstrated the capacity to alter the degree of steatosis following a two-hour treatment period, as evidenced by a notable reduction in liver tissue triglyceride and cholesterol levels (*P* < 0.05). Additionally, p-AMPK levels in liver tissue were significantly increased in the HOPE + AICAR group (*P* < 0.05). The combination of HOPE and AMPK activators has been shown to reduce the degree of steatotic liver grafts in a relatively short time, significantly reduce reperfusion injury, and improve liver function. This study contributes to the existing body of knowledge on mechanical perfusion in primate models, addressing a previously identified gap in the literature.

## Introduction

Liver transplantation is currently the most effective treatment for patients with end-stage liver disease and hepatocellular carcinoma. Living-donor liver transplantation has emerged as a crucial component of organ transplantation, with a notable proportion of living-donor liver transplantations in children^1^. In recent years, metabolic-associated fatty liver disease (MAFLD) has emerged as a significant global health concern, with the expansion of the MAFLD population contributing to an annual increase in the proportion of nonalcoholic steatotic liver donors for pediatric living liver transplantation^2–4^. However, because steatotic liver transplantation has been identified as an independent risk factor for post-implantation graft failure, many programs are reluctant to use steatotic livers^4,5^. Existing tests are unable to accurately assess the degree of hepatic steatosis preoperatively, and the presence of heavier steatotic livers may result in a poor prognosis.

Despite the significant reduction in the cold preservation time of donor livers in living liver transplantation, steatotic livers may still result in serious complications and poor prognosis after transplantation. This is because of the increased susceptibility of steatotic livers to ischemia-reperfusion(IR) injury^5,6^. Consequently, there is a pressing need to conduct further research and identify more effective methods of organ preservation to mitigate steatotic liver injury during ischemia, enhance organ utilization, and improve graft prognosis.

Perfusion preconditioning of transplanted livers represents a promising opportunity to obtain more organs for transplantation. Hypothermic Oxygenated Perfusion (HOPE) is a novel machine perfusion technique recently established to help attenuate (eliminate) IR injury^7–9^. It has been demonstrated to have a protective effect on graft function after extended donor thermal ischemia in various animal models^10,11^, and has also shown a good protective effect in human experiments^12–16^. It has been demonstrated that the primary mechanism of HOPE involves the reduction of mitochondrial oxidative stress, thereby attenuating IR injury in grafts^10,17^. For mechanical perfusion preservation of steatohepatitis, recent studies have shown that HOPE is beneficial for steatohepatitic livers by preventing significant reperfusion injury^18^. However, there is a paucity of evidence in the literature suggesting that HOPE can attenuate steatotic liver and improve prognosis within a shorter time window of living organ transplantation. We have gained insights from normothermic mechanical perfusion that the combined effects of NMP combined with degreasing drugs to promote lipid metabolism through sustained mechanical perfusion cycling and other actions are beneficial in decreasing the degree of steatosis in the donor liver and improving pre-transplantation donor liver quality^19–21^. Concurrently, nonhuman primates are of unique value in biomedical research. There is a paucity of experimental studies on the efficacy of HOPE in primate models. Consequently, there is a need to explore the effects of HOPE on steatotic liver grafts within a shorter time window using a primate model.

Adenosine-activated protein kinase (AMPK) is a principal sensor of energy stress and plays a pivotal role in the adaptive response to decreased energy levels^22^. AMPK is capable of responding to a range of metabolic stresses including hypoxia, ischemia and oxidative stress^23^. AMPK activators have been demonstrated to be protective against IR injury in the heart and kidney^24^. Furthermore, animal studies have indicated that activation of the AMPK pathway can reduce hepatic IR injury by decreasing lactate accumulation in the liver tissue and reducing hepatocyte necrosis^25^. However, no study has addressed the combined use of AMPK activators in the HOPE perfusion platform.

The objective of this study was to investigate the effects of an AMPK activator combined with HOPE on oxidative stress, lipid metabolism, and IR injury in the left lateral lobe of the liver. This was achieved through ex vivo perfusion of the left lateral lobe of cynomolgus monkey liver transplantation.

## Methods

### Experimental animals

All experimental protocols involving nonhuman primates were approved by the Committee for the Care of Laboratory Animals of Xi’an Jiaotong University (Approval No. 20191278) and the Committee for the Care and Use of Animals of the Chun Institute for Biotechnology Development (Approval No. 201901). This study was conducted in accordance with the National Institutes of Health Guide for the Care and Use of Laboratory Animals (8th edition, 2011). Eighteen male cynomolgus monkeys (age ≥ 9 years, BMI > 30) were selected and housed in individual cages with ad libitum access to water and food. The monkeys were housed in temperature-controlled rooms (22–26 °C) with a 12-hour dark and light cycle. They were fed a non-alcoholic steatohepatitis (NASH) diet.

NASH diet was purchased from Beijing Keao Xieli Feed Co. Ltd. (http://www.keaoxieli.com). The monkey maintenance formula diet was formulated according to the Nutrient Requirements of Non-Human Primates (Knapka, J. Nutrient Requirements of non-human Primates: Second Revised Edition. Lab Anim. 2003). (32, 26. 10.1038/laban1103-26). Prior to commencement of the physical examination (body weight, waist circumference, and abdominal circumference) and liver biopsy, the monkeys were anesthetized with ketamine hydrochloride (10 mg/kg body weight). Waist circumference was measured at the midpoint between the last rib and iliac crest, while abdominal circumference was measured at the height of the umbilicus. For the liver biopsy, a Bard Magnum biopsy gun (Bard Biopsy Systems, Tempe, AZ, USA) loaded with a 17-gauge biopsy needle (Argon, Athens, TX, USA) was used under the guidance of an ultrasound system (Landwind, P09, Shenzhen, China). The collected liver tissues were stored in formalin or at -80 °C.

After 10 months on the NASH diet, blood samples were collected from monkeys and anesthetized with ketamine hydrochloride (15 mg/kg body weight). Subsequently, the animals were euthanized by bloodletting. The livers were excised rapidly, and the blood within them was flushed out by perfusion with phosphate-buffered saline (PBS) at 4 °C. This was followed by cold preservation or HOPE, according to the experimental grouping. Liver tissues collected at specific time points during the experiment were stored in liquid nitrogen or formalin.

### Grouping

In this part of the experiment, 18 monkeys were randomly divided into three groups of six monkeys each.

**Fig. 1:**
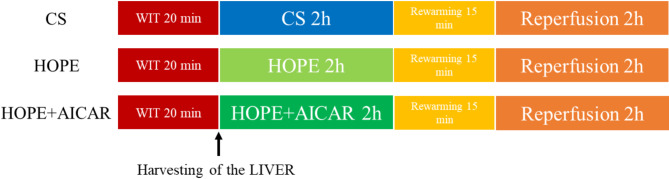
Experimental grouping and flow chart.

(1) Cold storage group (CS group): Following anesthesia and laparotomy, the livers were subjected to 20 min of thermal ischemia, after which they were obtained and placed in organ preservation containers containing the University of Wisconsin solution (UW solution) and stored statically for 2 h at 4 °C. The livers were rewarmed at room temperature for 15 min. After a 5-minute interval, the organs were subjected to a 2-hour ex vivo 37 °C incubation period. Subsequently, the organs were reinfused for 2 h at 37 °C in vitro.

(2) Hypothermic oxygenated perfusion (HOPE) group: After anesthesia and laparotomy, the liver was subjected to thermal ischemia for 20 min. The liver was obtained and placed in an organ preservation container containing 500 ml of University of Wisconsin solution (UW solution) at 4 °C for 2 h of HOPE. The livers were rewarmed at room temperature for 15 min. After a 5-minute interval, the organs were subjected to a 2-hour ex vivo 37 °C incubation period. Subsequently, the organs were reinfused for 2 h at 37 °C in vitro.

(3) Hypothermic oxygenated perfusion + AICAR group (HOPE + AICAR group): Following anesthesia and open abdomen, the liver underwent thermal ischemia for 20 min, after which the liver was obtained and placed in an organ preservation container containing 500 ml of the University of Wisconsin solution (UW solution) at 4 °C, and AICAR (MedChem, HY-13417) was added. AICAR was added to the preservation solution to achieve a final concentration of 1 mM. The solution was subjected to HOPE for 2 h. The livers were rewarmed at room temperature for 15 min. After a 5-minute interval, the organs were subjected to a 2-hour ex vivo 37 °C incubation period. Subsequently, the organs were reinfused for 2 h at 37 °C in vitro. The details of this procedure are shown in Fig. 1.

### Modelling of HOPE

A hypothermic oxygenated perfusion system was set up, comprising an ice bag, organ box, peristaltic pump, perfusion tubing, small-animal membrane oxygenator, 100% oxygen, thermometer, and perfusion fluid (UW fluid). Following removal of the liver, the organ was placed in an organ cassette with 500 ml of 4 °C UW fluid. An ice pack was placed within the organ cassette and the temperature of the perfusate was monitored using a thermometer to maintain a temperature of 4–10 °C. One end of the perfusion system was placed at the bottom of the organ box, and the peristaltic pump was activated to facilitate the flow of perfusion liquid through the membrane oxygenator (connected to 100% oxygen) and outflow perfusion tube. This process also involves evacuation of air bubbles from the perfusion system. The outflow perfusion tube was then connected to the hepatic portal vein perfusion tube to initiate the perfusion. During the perfusion process, fluid flows from the inflow perfusion tube, situated at the base of the organ cassette, into the oxygenator for full oxygenation. This is followed by flow from the portal vein into the liver for perfusion and finally from the inferior hepatic vena cava into the organ cassette, forming a dynamic process. The specific parameters of perfusion were as follows: perfusion flow: 300 ml/min, oxygen flow: The perfusion flow rate was 1 L/min, the total amount of perfusate in the entire circulatory system was 2 L, and the HOPE time was 2 h.

### Modelling of normothermic reperfusion

The normothermic reperfusion system can simulate pathophysiological conditions that occur after blood recirculation of the donor liver into the donor during liver transplantation. The perfusion system is analogous to the HOPE perfusion system, with the exception that the organ cassette is situated within a thermostatic water bath and the perfusate for reperfusion is constituted by autologous blood diluted in saline. This equates to 500 ml of diluted blood (50% The perfusate for reperfusion is composed of autologous blood diluted in saline (500 ml), 2 ml of 5% sodium bicarbonate solution, 10 ml of hydrocortisone, 2 µL of insulin, 0.2 g of cefazolin sodium for injection, and 5000 U of heparin sodium. The temperature of the perfusate during maintenance perfusion was maintained at 37 ± 0.5 °C, with the other equipment and connections remaining unchanged. Following the completion of the liver HOPE preservation procedure, the organ was maintained at room temperature for 15 min to facilitate rewarming. The reperfusion system was then activated to eliminate air bubbles in the pipeline. The outflow perfusion tube was subsequently connected to the hepatic portal vein perfusion tube to initiate normothermic perfusion. During the perfusion process, visible liquid was removed from the inflow perfusion tube and placed at the bottom of the organ cassette to enter the oxygenator for full oxygenation. It then flowed into the liver from the portal vein to perfuse and finally flowed out of the hepatic inferior vena cava and into the organ cassette, forming a dynamic process. The specific parameters of perfusion were as follows: perfusion flow: 300 ml/min, oxygen flow: 1 L/min, and reperfusion time at room temperature was 2 h. During the perfusion period, the perfusion flow was gradually stabilised at 300 ml/min from low to high to prevent the liver from rupturing due to the instantaneous overpressure of the perfusion pressure.

### Specimen collection

During the perfusion process, morphological alterations of the liver were meticulously observed to avoid the occurrence of edema rupture. Perfusion effluents were collected at the following time points: before and after 2 h of HOPE; before and after two hours of simulated reperfusion. Subsequent to the conclusion of the perfusion procedure, the liver was harvested for either fixation or cryopreservation.

### Biochemical examination

A sample of perfusate was collected and 1.5 ml of perfusate was used to measure alanine aminotransferase (ALT), aspartate aminotransferase (AST) and lactate dehydrogenase (LDH) using a fully automated biochemistry analyser, Chemray 240 (Rayto). The perfusate and liver tissue malondialdehyde (MDA) levels were quantified in accordance with the instructions provided with the malondialdehyde (MDA) test kit (Nanjing Jianchen, A003-1). The liver lipid levels were determined using the triglyceride assay kit and total cholesterol assay kit instructions (Changchun Huili). The quantification of liver ATP levels was conducted by means of an ATP content assay kit (Wuhan Servicebio).

### Histological staining

Liver biopsy sections were stored in 4% neutral formalin buffer for a period of 24 to 48 h, after which they were embedded in paraffin. Three-micrometre-thick tissue sections of the paraffin blocks were taken from each group of animals and stained with haematoxylin and eosin (HE). The HE staining was employed to qualitatively evaluate the gross morphology of the liver and the histological alterations of the liver following HOPE. Three-micrometre-thick frozen sections of each group of animals were stained with oil red O staining, with the objective of qualitatively evaluating the lipid alterations of the liver following HOPE.

### ELISA

The perfusate and liver were collected and the concentrations of tumour necrosis factor-α (TNF-α), interleukin 6 (IL-6), lactic acid (Lactic Acid), 8-hydroxydeoxyguanosine (8-OHdG), and toll-like receptor (TLR4) were determined using an enzyme-linked immunosorbent assay (ELISA) kit (Shanghai Enzyme-linked).

### Western blot analysis

The western blotting method was employed to detect AMPK and p-AMPK protein expression. Total protein was extracted from liver tissue using the RIPA lysate, and the total protein concentration was determined using the BCA method (Shanghai beyotime). The protein was then denatured at 100 °C for 10 min. SDS-PAGE gels were prepared, and the protein samples (10 µg) were electrophoresed for 1.5 h, transferred to the membrane for 1.5 h, and blocked for 2 h. The primary antibody (CST) was incubated with the corresponding dilution of the containment solution on the PVDF membrane at 4 °C overnight. For the incubation of the secondary antibody, the corresponding HRP-labelled secondary antibody was diluted with the containment solution, and the PVDF membrane was incubated with the secondary antibody on a shaker at 37 °C for 2 h. For the colour development, the enhancement solution in E The CL reagent was combined with the peroxidase solution in a 1:1 ratio, and the resulting working solution was added to the PVDF membrane in a dropwise manner for a period of several minutes. Following this, the development, fixation, washing, and scanning were conducted in sequence. The grey values were analysed using the BandScan software, with GAPDH serving as the internal reference.

### Statistical analysis

The data were statistically analyzed using GraphPad Prism software (version 8.0), and all data are expressed as mean ± standard deviation (mean ± SD). The unpaired t-test was employed for the comparison of two groups, while one- or two-way ANOVA followed by Tukey’s test was used for multiple comparisons. Statistical significance was set at *P* < 0.05.

## Results

1. The combination of HOPE and AICAR has demonstrated to reduce the damage to liver tissue associated with DCD.

**Fig. 2:**
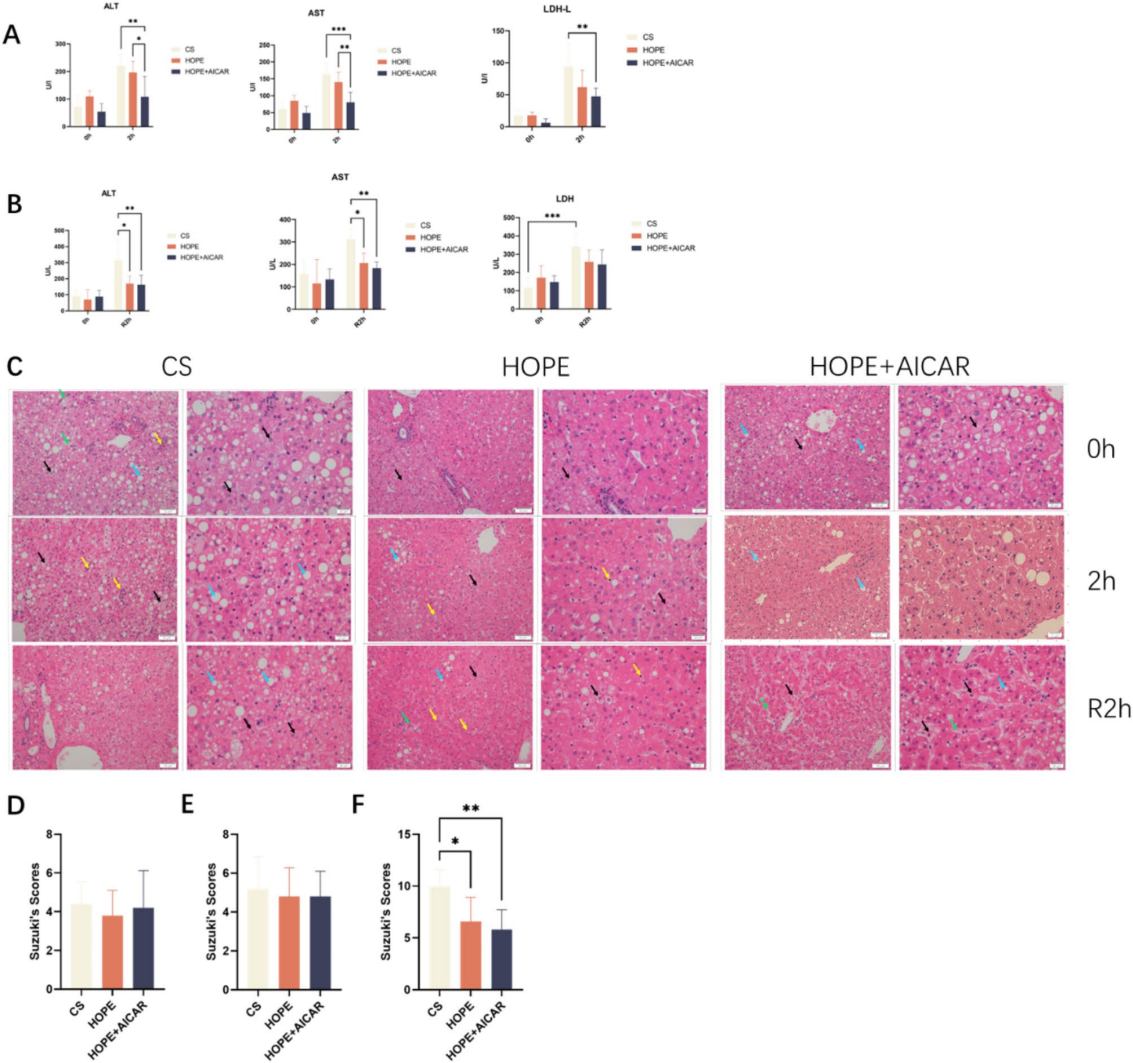
The combination of HOPE and AICAR has been demonstrated to reduce the damage to liver tissue caused by DCD. (**A**) Analysis of AST, ALT and LDH levels in perfusate of the HOPE model; (**B**) analysis of AST, ALT and LDH levels in perfusate of the normothermic reperfusion model; (**C**). Representative images of HE staining of hepatic tissue Sections, scale bars = 50 μm and 20 μm; (**D**–**F**) Analysis of Suzuki score of the degree of liver injury. (**D**–**F**) represent 0 h, 2 and R2h, respectively; all data are expressed as mean ± SD (*n* = 6 per group), *P < 0.05. The liver tissue exhibited steatosis, as evidenced by the presence of blue arrowheads. Cytoplasmic laxity of certain hepatocytes is observable (green arrows). The number of inflammatory cells within the hepatic sinusoids increased, with punctate infiltration of individual inflammatory cells observed (yellow arrows). Additionally, individual hepatocytes exhibited necrosis (black arrows).

After 10 months on the NASH diet, we confirmed the presence of NASH and its progression in our model animals using liver biopsy and histological assessment of NAS. Specific results can be found in a published article (10.1016/j.cmet.2023.03.013). To ascertain whether the combination of HOPE and AICAR could mitigate DCD donor liver injury in cynomolgus monkeys, we initially examined the levels of AST, ALT, and LDH in HOPE perfusate. The results demonstrated that following 2 h of static cold preservation, the levels of AST, ALT, and LDH in the perfusate of the CS group were markedly elevated. Compared with the CS group, the levels of AST, ALT, and LDH in the perfusate of the HOPE and HOPE + AICAR groups were significantly reduced after 2 h of hypothermic oxygenated perfusion, with the greatest reduction observed in the HOPE + AICAR group (Fig. 2A), indicating that HOPE can mitigate the damage to DCD donor livers during ex vivo preservation. Furthermore, AST, ALT, and LDH levels in the perfusate were analyzed in the normothermic reperfusion model. The results demonstrated that AST, ALT, and LDH levels were significantly elevated in the three groups following normothermic reperfusion, particularly in the CS group. In comparison to the CS group, AST, ALT, and LDH levels were reduced in the HOPE and HOPE + AICAR groups (Fig. 2B). However, LDH levels did not differ statistically significantly between the groups. To further verify these results, we performed HE staining and Suzuki scoring on the liver tissues of each group. Following reperfusion, liver tissue damage was significantly aggravated in the CS group, as evidenced by a more disorganized arrangement of the tissue hepatocyte cords, narrowing of the hepatic sinusoids, swelling of the hepatocyte cytosol, and a large number of hepatocytes with steatosis. The number of inflammatory cells increased and there was a greater incidence of hepatocyte necrosis. However, compared with the CS group, HOPE significantly attenuated the histological injury of the DCD donor liver (Fig. 2C–F), with reduced inflammatory infiltration and a small amount of hepatocyte necrosis, especially in the HOPE + AICAR group. These results demonstrated that HOPE could mitigate liver tissue injury in DCD in cynomolgus monkeys, particularly when AICAR was included in the perfusate.

2. The combination of HOPE and AICAR has demonstrated to reduce hepatic lipid accumulation in cynomolgus monkeys with DCD.

**Fig. 3:**
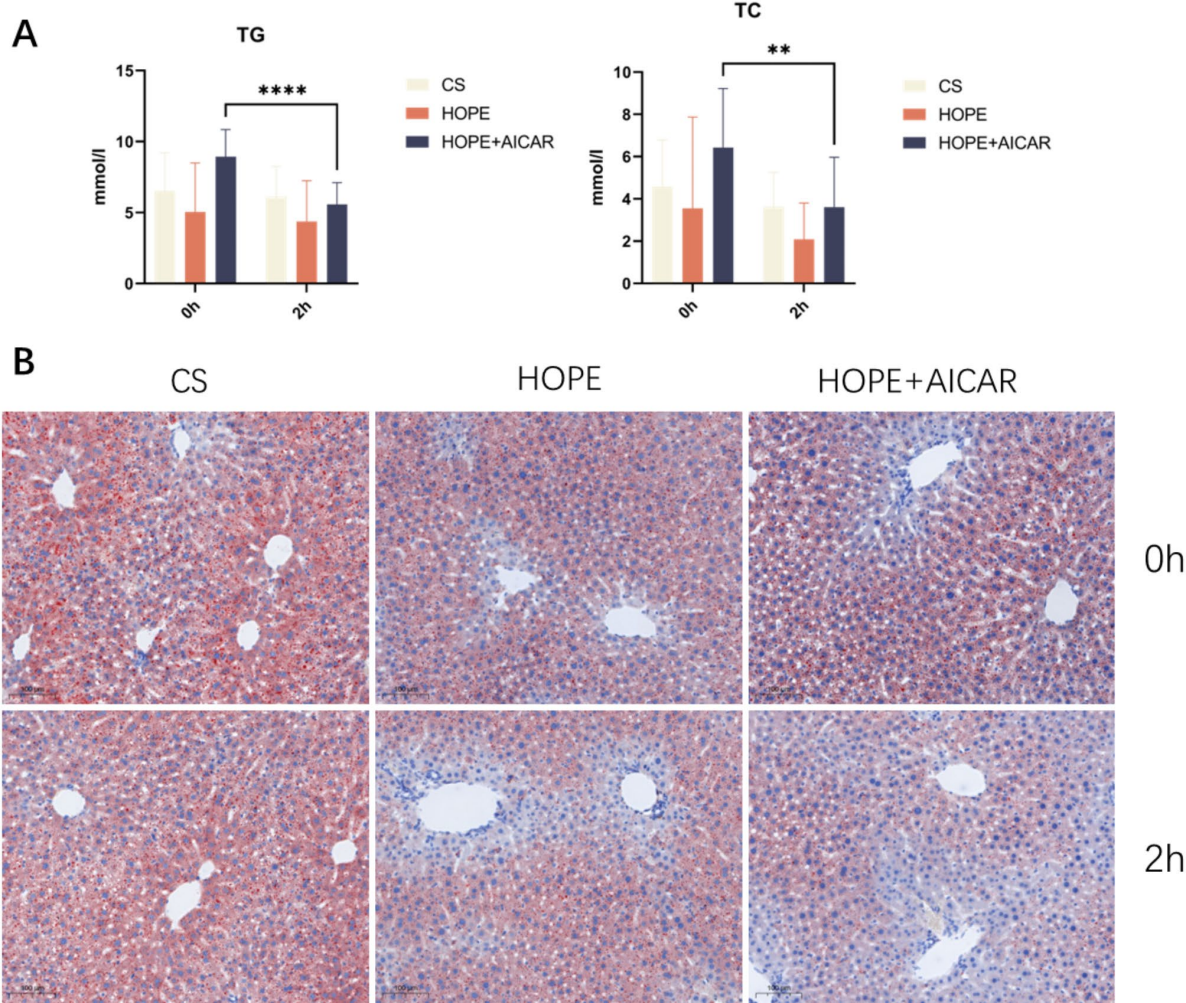
The combination of HOPE and AICAR has been demonstrated to attenuate hepatic lipid accumulation in cynomolgus monkeys with DCD. (**A**) Analysis of TG and TC content of liver tissue homogenates; (**B**) Representative images of HE staining of liver tissue sections, scale bar = 100 μm; all data are expressed as MEAN ± SD (*n* = 6 per group), *P < 0.05.

HOPE, as a new organ preservation option, regulates the energy metabolism of organs. Furthermore, adenosine-activating enzyme (AMPK) is a principal sensor of energy stress and plays a pivotal role in the regulation of energy levels. To investigate the effects of HOPE and additional addition of AICAR on hepatic lipid accumulation in DCD and to ascertain whether they could attenuate hepatic lipid accumulation, we initially examined the levels of triglyceride and total cholesterol in liver tissue homogenates. Triglycerides represent a significant component of fat within liver cells, with elevated levels correlating positively with the severity of fatty liver disease. Similarly, total cholesterol constitutes an essential component of fat within liver cells. As the progression of fatty liver disease advances, total cholesterol levels concomitantly increase. The results demonstrated that the levels of triglycerides and total cholesterol were unchanged in the CS and HOPE groups, whereas the HOPE + AICAR group exhibited a significant decrease in triglyceride and total cholesterol levels in liver tissue homogenates after hypothermic oxygenated perfusion. The results indicated that HOPE was effective in reducing lipid accumulation in the liver tissue after the addition of AICAR (Fig. 3A). Furthermore, Oil Red O staining of liver tissues in each group demonstrated that there was no significant difference in steatosis between the CS and HOPE groups, while lipid accumulation was attenuated in the HOPE + AICAR group (Fig. 3B). These results demonstrated that HOPE perfusion in steatotic livers did not alter the degree of steatosis prior to implantation. Conversely, the combination of HOPE and AICAR attenuated the accumulation of liver lipids in the DCD livers of cynomolgus monkeys.

3. The combination of HOPE and AICAR has demonstrated to reduce oxidative stress-related injury to the liver with DCD.

**Fig. 4:**
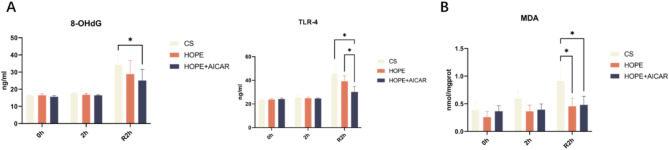
The combination of HOPE and AICAR attenuates oxidative stress injury in DCD liver. (**A**) Analysis of 8-OHdG and TLR-4 content in liver tissues; (**B**) Analysis of MDA content in liver tissues; all data are expressed as mean ± SD (*n* = 6 per group), *P < 0.05.

Hepatic oxidative stress was evaluated by detecting 8-OHdG and TLR4 expression in the liver after reperfusion. 8-OHdG has been identified as a product of oxidative DNA damage, and an elevated level of this compound has been shown to reflect the degree of oxidative stress.TLR-4, a pattern recognition receptor, has been demonstrated to be closely related to the severity of liver injury with regard to both its expression level and activity. We first detected the release of 8-OHdG and activation of TLR-4 in liver tissues under HOPE and normothermic reperfusion in each group. The results demonstrated that cold preservation significantly augmented oxidative stress in the liver, as evidenced by the augmented release of 8-OHdG and activation of TLR-4 after reperfusion. HOPE and HOPE + AICAR treatment prevented oxidative stress after reperfusion, with a reduction in nuclear DNA oxidation and an improvement in the activation of TLR-4, which was particularly evident in the HOPE + AICAR group (Fig. 4A). Furthermore, MDA, an end product of lipid peroxidation, has been identified as a marker of the degree of lipid peroxidation. the MDA content was significantly higher in the liver tissue of the CS group. However, HOPE and the combination of HOPE and AICAR treatment significantly reduced MDA content in liver tissues compared to CS (Fig. 4B). In conclusion, the combination of HOPE and AICAR was effective in reducing hepatic oxidative stress injury in DCD.

4. The combination of HOPE and AICAR has demonstrated to attenuate the hepatic inflammatory response in DCD.

**Fig. 5:**
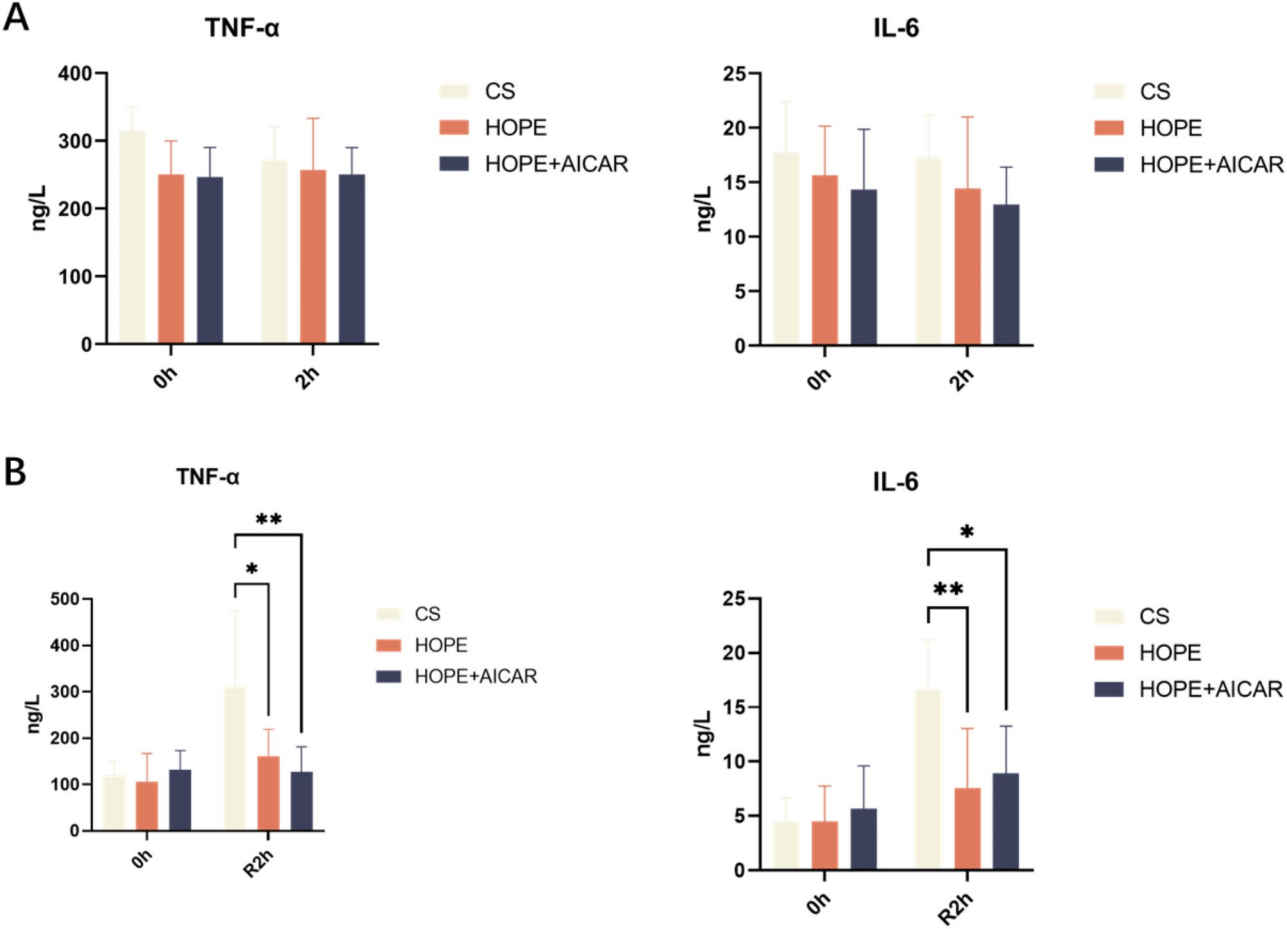
The combination of HOPE and AICAR attenuates hepatic inflammatory response in DCD. (**A**) Analysis of TNF-α and IL-6 levels in the perfusate of the HOPE model; (**B**) analysis of TNF-α and IL-6 levels in the perfusate of the normothermic reperfusion model; all data are expressed as mean ± SD (*n* = 6 per group), *P < 0.05.

The study’s findings indicated that the levels of pro-inflammatory cytokines TNF-α and IL-6 in the perfusate of the CS group exhibited a significant increase after 2 h of reperfusion in comparison with the 0-hour baseline. Nevertheless, the levels of TNF-α and IL-6 were significantly lower in both the HOPE group and the combination of HOPE and AICAR groups than in the CS group after reperfusion. This indicated that HOPE, as well as the combination of HOPE and AICAR, reduced the production of pro-inflammatory cytokines in DCD livers (Fig. 5A,B).

5. The combination of HOPE and AICAR has shown to improve energy metabolism in DCD livers.

**Fig. 6:**
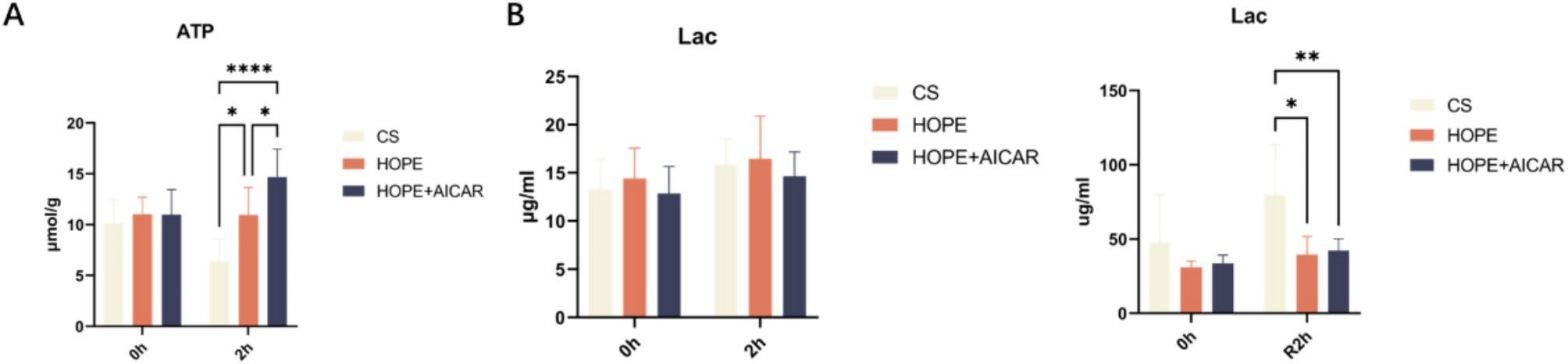
The combination of HOPE and AICAR improves energy metabolism in DCD livers. (**A**) Analysis of ATP content in liver tissue; (**B**) Analysis of lactate levels in the perfusate of the HOPE model and the perfusate of the ambient reperfusion model; all data are expressed as MEAN ± SD (*n* = 6 for each group), *P < 0.05.

To ascertain whether the combination of HOPE and AICAR could enhance the energy metabolism of hepatic tissue, we examined the adenosine triphosphate (ATP) level in the liver tissue and the lactate content in the perfusate in each group. The results demonstrated that the ATP level was significantly higher in the HOPE + AICAR group than in the CS group (Fig. 6A), likely because of the continuous oxygen supply to the liver, which facilitated ATP synthesis. In comparison with the CS group, the lactate concentration in the perfusate of the HOPE and HOPE + AICAR groups demonstrated a significant reduction after 2 h of low-temperature oxygen-carrying mechanical perfusion during the reperfusion period (Fig. 6B). These findings indicate that the combination of HOPE and AICAR can enhance the energy metabolism of the DCD liver by augmenting the ATP content and reducing lactate production.

6. The combination of HOPE and AICAR has demonstrated to enhance the expression of p-AMPK protein in liver tissue.

**Fig. 7:**
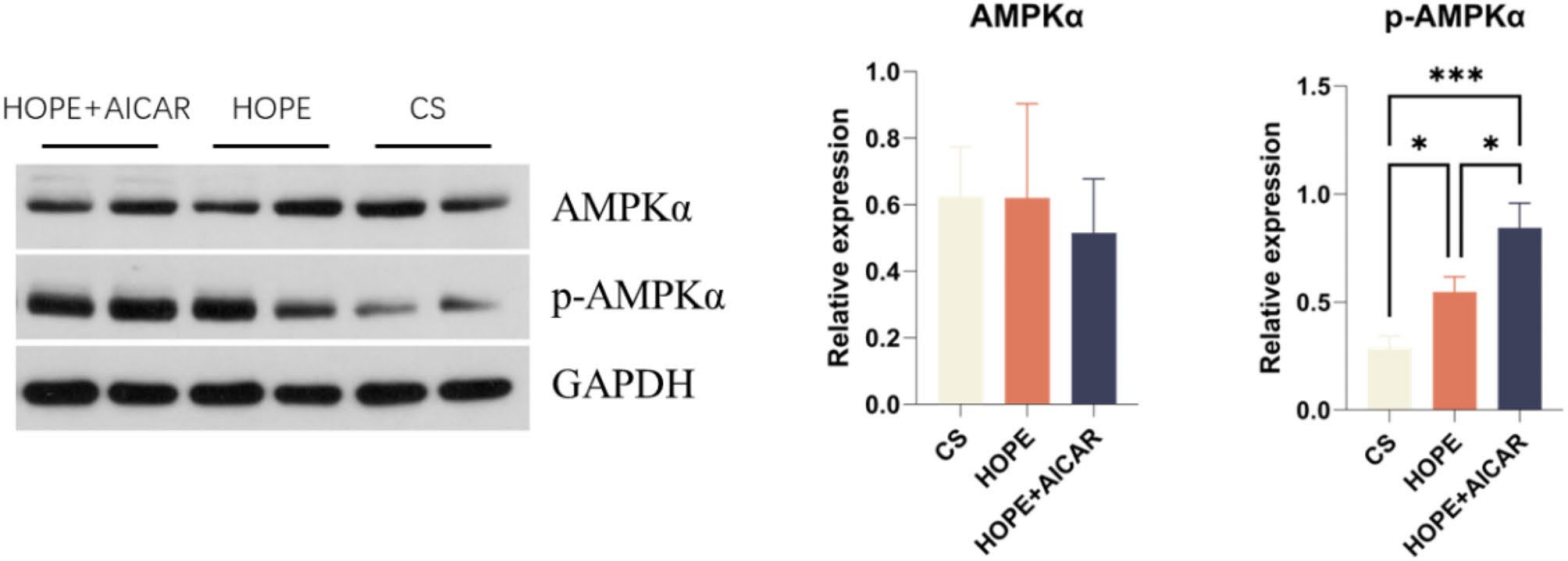
The combination of HOPE and AICAR increased the expression level of p-AMPK protein in liver tissue. Expression of AMPK and p-AMPK protein levels in the liver of each group. Representative blots from each group are shown and quantification of band intensity (normalised to GAPDH) was performed. All data are expressed as mean ± SD (*n* = 6 per group), *P < 0.05.

Consequently, we conducted a protein blotting assay to examine the changes in AMPK and p-AMPK protein expression in liver tissue. The results demonstrated that p-AMPK protein expression in liver tissue was significantly higher in the HOPE + AICAR group than in the other two groups (*P* < 0.05) (Fig. 7).

## Discussion

With the increasing maturity and improvement of organ transplantation technology, liver transplantation has become the most effective treatment for end-stage liver disease and is widely used worldwide^1^. Furthermore, living-donor liver transplantation has become an important way to save children with end-stage liver disease. The current method for graft preservation is cryopreservation.

However, there are still issues with energy depletion of the graft during cold storage in preservation solutions (cold ischemia), oxidative stress, and inflammatory responses. The response of the graft recipient to revascularization (reperfusion) can result in unavoidable IR injury. IR injury is associated with delayed graft function and primary graft failure and represents a significant clinical problem following liver transplantation^26^. In addition, the use of marginal donors, defined as donors with hepatic steatosis, is becoming increasingly prevalent in liver transplantation clinics^4^. Steatotic liver grafts are more susceptible to IR injury, which increases the risk of IRI and primary graft nonfunction, as well as recipient death^27^. Therefore, techniques to improve the function of steatotic liver grafts have become a popular research topic worldwide. The primary objective of this study was to investigate methods of improving graft function during the brief period between procurement of the donor liver for living liver transplantation and transplantation surgery. In this study, we employed non-primate cynomolgus monkeys to construct a steatotic liver donor model. We developed an ex vivo HOPE model and preserved the ex vivo donor liver via perfusion for 2 h. During this period, we innovatively added the AMPK agonist AICAR to enhance the preservation effect of perfusion. Subsequently, an isolated normothermic reperfusion model was established to simulate the reperfusion process following restoration of blood flow during transplantation.

Over the past decade, several innovative preservation strategies have been developed with the aim of “saving” marginal livers before transplantation^28,29^. HOPE, as an alternative to static cold preservation, is expected to play an important role in expanding the standards of donor organ preservation^30^. Yoshikawa et al. mechanically perfused DCD porcine livers with cryo-oxygenation for 4 h. The results of this study demonstrate that the experimental group exhibited a significant reduction in hepatocellular necrosis, a low level of serum markers of injury, and an increase in bile secretion. These findings confirm the superiority of short term cryo oxygenation mechanical perfusion over purely static cold preservation^31^. In 2021, van Rijn et al. conducted a multicenter, randomized controlled trial. The HOPE method for preserving DCD livers demonstrated a 68% relative reduction in the risk of non-anastomotic biliary strictures (NABS) after liver transplantation and a significant reduction in the risk of multiple post-liver transplantation complications^32^. In contrast, for steatotic liver grafts, IR injury is more severe because of hepatocyte microcirculatory damage, reactive oxygen species production, and lipid peroxidation. Kron analyzed the effects of HOPE on steatotic liver grafts using a rodent model of in situ liver transplantation (OLT). The results showed that HOPE after cold preservation of steatotic livers prevented significant reperfusion injury and improved the graft function. This finding suggests that HOPE is a safer and more reliable method for donor liver preservation^18^. Panconesi et al. demonstrated that HOPE can save high-risk DCD livers by improving mitochondrial injury^33^. Non-human primates (NHP) exhibit a high degree of similarity to humans with regard to a number of key biological and behavioral traits, including physiology, cognitive ability, neuroanatomy, social complexity, reproduction, and development^34,35^. They share 75–98.5% homology with human genetic material^36^, which plays an important role in basic and translational biomedical research. Accordingly, we employed the cynomolgus monkey as a primate model with the objective of partially addressing the gap in the utilization of primate models in the investigation of mechanical perfusion. However, for living liver transplantation, donor livers have a limited preservation time window. It remains unclear whether it is possible to improve the quality of the donor liver within a short period of time. In this study, we used HOPE to preserve the donor liver within a short period and successfully attenuated reperfusion injury, demonstrating the effectiveness of applying HOPE within a short period of time.

The poor prognosis of grafts is due to depletion of adenosine triphosphate (ATP) during cold preservation. A continuous supply of oxygen and adenosine can maintain high ATP levels in the preserved tissues, thereby reducing oxidative damage and metabolic stress. Therefore, ATP is important for maintaining the function of marginal donor livers with varying degrees of ischemic injury and continuous ATP depletion^37,38^. Consequently, ATP plays an important role in functional repair of the donor liver, particularly in the context of improved steatotic liver preservation. Lüer et al. demonstrated that the use of 100% oxygen-concentrated perfusate significantly reduced transaminase release during perfusion and significantly increased the activation of the adenylate-activated protein kinase (AMPK) remedial synthesis pathway and upstream protein kinase A^39^. These results indicate that AMPK is a key enzyme in the regulation of energy metabolism and plays a critical role in adaptive responses to declining energy levels, including hypoxia. AMPK activation has been shown to reduce body weight and hepatic fat content as well as improve lipid levels and insulin resistance^40,41^. Furthermore, AMPK is sensitive to intracellular oxidative stress and can be regulated by affecting the mitochondrial function. Its mechanism of inhibiting inflammatory responses may be achieved by blocking signalling pathways, such as NF-xB, MAPK, and JAK-STAT^42,43^.

The results of numerous experimental studies indicate that HOPE may offer greater advantages over cold preservation for steatotic liver grafts when the preservation window is shorter^44,45^. The addition of the AMPK agonist AICAR may produce more favorable results in the preservation of steatotic liver grafts. In this study, a combination of HOPE and AICAR was employed as the primary method for the preservation of steatotic liver grafts. These results demonstrate that this modality effectively reduced liver damage markers. Concurrently, the combination of HOPE and AICAR reduces oxidative stress in the liver tissue and the expression of inflammatory factors during reperfusion. Furthermore, it is of significant importance to note that the combination of HOPE with AICAR enhanced the energy metabolism of the steatosis liver, which could mitigate the impact of lipid accumulation in the liver tissue more effectively than HOPE alone. This is crucial for improving the microcirculation of hepatocytes and protecting damaged hepatocytes. To further verify the mechanism of action of improving the steatotic liver, the expression levels of AMPK and p-AMPK proteins in liver tissues were measured. The combination of HOPE and AICAR significantly activated AMPKα, indicating that the protective effect of this modality on the steatotic liver may be attributed to the activation of the AMPK signalling pathway.

However, this study has some limitations. Firstly, the transplantation model is of significant importance. Unfortunately, we were unable to use the monkey liver transplantation model due to limitations with the experimental conditions. Secondly, during the process of isolated mechanical perfusion, various perfusion parameters were not observed and adjusted in real time due to various conditions. Thirdly, this study initially explored the improvement effect of the combination of HOPE and AICAR on steatosis in the liver, and did not carry out in-depth exploration and research on the underlying mechanisms.

## Conclusion

The combination of HOPE and AMPK activators has been shown to reduce the degree of steatotic liver grafts in a relatively short time, significantly reduce reperfusion injury, and improve liver function. The protective mechanism is likely to be attributed to activation of the AMPK pathway, mitochondrial repair, and prevention of oxidative stress after reperfusion during AMPK activation combined with HOPE. This study contributes to the existing body of knowledge on mechanical perfusion in primate models, addressing a previously identified gap in the literature.

## Data Availability

The datasets generated and/or analysed during the current study are not publicly available due privacy concerns but are available from the corresponding author on reasonable request.
